# Mechanical perfusion in brain banking: methods of assessment and relationship to the postmortem interval

**DOI:** 10.17879/freeneuropathology-2025-8880

**Published:** 2025-10-21

**Authors:** Macy Garrood, Alicia Keberle, Gabriel A. Taylor, Emma L. Thorn, Claudia De Sanctis, Kurt Farrell, John F. Crary, Jordan S. Sparks, Andrew T. McKenzie

**Affiliations:** 1 Apex Neuroscience, Salem, Oregon, USA; 2 Friedman Brain Institute, Departments of Pathology, Neuroscience, and Artificial Intelligence & Human Health, Icahn School of Medicine at Mount Sinai, New York, New York, USA; 3 Neuropathology Brain Bank & Research Core and Ronald M. Loeb Center for Alzheimer's Disease, Icahn School of Medicine at Mount Sinai, New York, New York, USA

**Keywords:** Brain banking, Mechanical perfusion, Perfusion fixation, Preservation quality, No-reflow phenomenon

## Abstract

The mechanical perfusion of solutions through the cerebrovascular system is critical for several types of postmortem research. However, achieving consistently high-quality perfusion in this setting is challenging. Several previous studies have reported that longer postmortem intervals are associated with decreased perfusion quality, but the mechanisms and temporal progression of perfusion impairment are poorly understood. In this study, we describe our experience in developing a protocol for *in situ* perfusion of the postmortem brain in human whole-body donors (n = 77). Through the evaluation of different approaches, we found that cannulation of the internal carotid arteries combined with clamping of the vertebral arteries allows targeted perfusion of the brain. We evaluated perfusion quality through three complementary methods: gross anatomical appearance, CT imaging, and histology. These quality assessment measures were partially correlated across donors, indicating that they offer complementary perspectives on perfusion quality. Correlational analysis of our cohort of banked brains confirms that perfusion quality decreases as the postmortem interval increases, with a heterogenous pattern across brain regions. Our findings provide data for optimizing brain banking protocols and suggest future directions for investigating the mechanisms of postmortem perfusion impairment.

## Abbreviations

**ACA** - Anterior cerebral artery, **BBB** - Blood-brain barrier, **CB** - Cerebellum, **CSF** - Cerebrospinal fluid, **CT** - Computed tomography, **FCTX** - Frontal cortex, **H&E** - Hematoxylin and Eosin, **ICC** - Intra-Class Correlation, **IJV** - Internal jugular vein, **MCA** - Middle cerebral artery, **NBF** - Neutral Buffered Formalin, **OCTX** - Occipital cortex, **PCA** - Posterior cerebral artery, **PMI** - Postmortem Interval, **TCTX** - Temporal cortex, **THAL** - Thalamus, **TWM** - Temporal white matter, **WSI** - Whole slide image.

## Introduction

In postmortem brain research, mechanical perfusion i.e. the delivery of solutions through blood vessels using external pressure from pumps, gravity systems, or syringes rather than the natural heartbeat has proven valuable for several specialized research applications. These include the delivery of fixatives for tissue preservation (Brown, 1939; McFadden et al., 2019), latex or other compounds for vascular mapping (Duvernoy et al., 1981; Alvernia et al., 2010), radio-opaque substances for angiography (Turkoglu et al., 2014), and detergents or other chemicals for brain clearing (Zhao et al., 2020). Mechanical perfusion can be performed with the brain both *in situ* (inside of the skull) and *ex situ* (removed from the skull). It can be an effective way to deliver chemicals throughout large organs such as the brain because it utilizes the existing vascular network to distribute substances widely. However, even in ideal, controlled laboratory animal settings, mechanical perfusion remains a challenging problem, often leading to heterogenous perfusion across the brain (Bodian, 1937; Cahill et al., 2012; Schwarzmaier et al., 2022). It is even more challenging to achieve consistently high-quality perfusion in the postmortem human brain, because of the diverse physiological changes that occur in the agonal and postmortem periods. We define perfusion impairment as the observed inability to mechanically perfuse tissue as typically occurred during life. Perfusion impairment shares many similarities with the clinical phenomenon of "no-reflow", wherein there is a failure of microvascular perfusion after a period of ischemia, despite restoration of arterial flow (Kaul et al., 2022). While no-reflow has been extensively studied in the context of reperfusion after stroke and myocardial infarction, the phenomenon of perfusion impairment in the postmortem brain is not yet well understood.

Numerous studies have reported that the ability to drive solutions through the brain’s blood vessels decreases with longer intervals between the cessation of blood flow and the initiation of perfusion (McFadden et al., 2019; Mignucci-Jiménez et al., 2024). That perfusion of the postmortem body in general is impaired by a longer period of ischemia has been known for centuries. For example, in the 18th century, William Hunter recommended initiating perfusion with preservative solutions for anatomical studies within eight hours after death in the summer and within 24 hours after death in the winter (Bryant, 2003, p. 537). Studies on the brain in particular have reported that perfusion quality begins to deteriorate just 5 minutes after death (Bodian, 1936; Karlsson and Schultz, 1966). However, the precise rates of this decline are unclear, especially for human brain specimens (McFadden et al., 2019). The progression of perfusion impairment can also be heterogeneous across brain regions. For example, one study found that after 10–15 minutes of ischemia, perfusion defects in the rabbit brain developed in a patchy distribution, with some areas showing complete perfusion failure while adjacent regions remained perfusable (Ames et al., 1968). The extent to which this heterogeneity of perfusion impairment is haphazard or regionally patterned is unclear, with some previous research finding a tendency for perfusion defects in certain regions (Ames et al., 1968). The regional heterogeneity could depend on multiple factors, including the relative degree of blood flow in those regions during life, the patterns of cerebrovascular pathology that developed prior to death, the route of mechanical perfusion, differences in postmortem edema, postmortem lividity, or gravitational effects (Du et al., 2022; Frigon et al., 2024).

Assessing the degree to which the perfused fluid actually traverses through the blood vessels throughout the brain – i.e., the quality of perfusion – also presents challenges. Investigators have approached the quality assessment problem in several ways. At the macroscopic level, perfusion quality can be evaluated on the basis of tissue color, firmness, swelling or shrinkage, consistency during cutting, and the clearance of blood from surface vessels (Jenkins et al., 1979; McFadden et al., 2019; Monroy-Gómez et al., 2020). However, reliance on surface appearance alone can be misleading, as it may not reflect successful perfusion of deeper brain regions. It has been reported that major vessels may be perfused but while the perfusate still fails to penetrate into the small vessels or most of the tissue itself (Palay et al., 1962). Assessment methods during the perfusion procedure include monitoring flow rates, venous return characteristics, changes in perfusion pressure, and ocular tension (Donckaster and Politoff, 1963; Schwarzmaier et al., 2022). Histological evaluation is generally considered the gold standard, but is resource-intensive when applied to large tissue areas. Specific histological metrics include the presence of red blood cells in vessels, vessel dilation, and perivascular space morphology (Scharrer, 1938; Palay et al., 1962; Wohlsein et al., 2013). Since slower preservation leads to more postmortem changes, the extent of autolysis can serve as an indirect proxy for perfusion quality (Frigon et al., 2022). Finally, some studies have added chemicals to the perfusate to aid in visualizing perfusion quality. For example, Garcia et al., 1977 used colloidal carbon as an electron-dense tracer, to directly visualize capillary perfusion via electron microscopy (Garcia et al., 1977). Neuroimaging approaches have also enabled non-destructive, three-dimensional assessment of perfusate distribution (Böhm, 1983). Despite this wide array of quality assessment methods, direct comparisons between them remain limited – particularly in the context of human brain banking, where imperfect perfusion is expected.

Although the mechanisms of postmortem perfusion impairment are not yet fully understood, insights can be drawn from adjacent fields. Mechanistic factors that contribute to perfusion impairment after temporary cerebrovascular ischemia include blood coagulation, vessel constriction, pericyte contraction, and cellular edema (Kaul et al., 2022). An important early source of parenchymal fluid accumulation after ischemia appears to be backflow from the cerebrospinal fluid (CSF). CSF begins to enter brain tissue within two to three minutes after circulatory arrest, with peak influx around 7 minutes (Du et al., 2022). This influx may be associated with pressure gradients created by the loss of blood pressure and coincides with the onset of anoxic spreading depolarization (Weed, 1914; Du et al., 2022). The early postmortem accumulation of fluid tends to occur in astrocyte processes, which become swollen, including around blood vessels and certain neurons (Krassner et al., 2023). These swollen astrocyte processes can externally compress capillaries, contributing to perfusion impairment and possibly helping to explain why perfusion failure develops more rapidly in the brain than in other tissues (Majno et al., 1967). Finally, blood-brain barrier (BBB) alterations during ischemia also contribute to perfusion impairment. One study found that BBB disruption, as indicated by extravasation, occurs rapidly during reperfusion following 30 minutes of global cerebral ischemia in mice (Ju et al., 2018). Notably, in this study, venules and capillaries were found to have the most leakage, with the leaky vessels were predominantly venules (48 %) and capillaries (25 %). Together, these mechanisms appear to act synergistically to progressively impair perfusion in the postmortem brain.

While the postmortem interval (PMI) is obviously a key factor affecting perfusion quality, several other donor characteristics can also significantly influence perfusion success as well. The agonal state – i.e. the physiological condition preceding death – appears to be particularly important. Prolonged agonal states can cause blood vessel inflammation, thrombus formation, and BBB disruption (Perry et al., 1982; Hardy et al., 1985; Hansma et al., 2015). As a result, donors with extended periods of hypoperfusion, hypoxia, or multi-organ failure may have more severe perfusion impairment for a given PMI compared to those who die suddenly. Advanced age and various disease processes can also affect perfusion quality through mechanisms such as atherosclerosis and altered vascular compliance, independently of the PMI. Donor use of anticoagulant medications such as heparin or apixaban prior to death is likely to decrease the extent of thrombus formation during the agonal phase and postmortem period. Environmental factors such as ambient temperature and humidity during the PMI likely also play a large moderating effect on the degree of perfusion impairment. The complex interplay between these factors – agonal state, age, disease history, medications, and environmental circumstances during the PMI – likely contributes to substantial variability in postmortem brain perfusion beyond the PMI alone.

In this study, we describe our methods for performing mechanical perfusion fixation of the postmortem brain and for assessing perfusion quality. After testing multiple methods for perfusion, we adopted an *in situ* approach on the isolated cephalon, involving cannulation of the internal carotid artery and clamping of the vertebral arteries. We evaluated perfusion quality using multiple modalities, including gross examination, neuroimaging, and histology. In our experience, perfusion fixation is of variable efficacy, with heterogeneity observed both across donor brains and across areas of the same brain. Because of our limited sample size and lack of systematic comparisons, we do not claim that our method for postmortem perfusion is superior to others. Instead, we provide data and practical insights that will help other investigators in optimizing their own methods of mechanical perfusion, whether for fixation or other purposes.

## Methods

### Whole body donation procedures

Anatomical whole-body donations were performed by a partner whole body donation organization operating under Oregon Health Authority regulations. All donations were processed after obtaining informed consent. The Apex Neuroscience Brain and Tissue Bank operates under an exemption determination issued by the Pearl Institutional Review Board (IRB) after submission of our protocols for review (Pearl IRB ID #2023-0260). The cohort was a convenience sample of whole-body donors with no specific exclusion criteria, other than confirmed or suspected transmissible disease such as HIV, hepatitis B/C, or prion disease. All cases where perfusion was performed were included in this data set, provided that data from at least one modality (gross examination, CT scan, or histology) were available to assess the quality of perfusion.

### Perfusion procedure

In our initial experiments, we used an anterior cervical approach with cannulation of the common carotid arteries or internal carotid arteries. Subsequently, the method of perfusion was adapted for use on isolated cephalon specimens, following neck dissection at the level of C4–C5. The cephalon was secured in a container filled with ice, aiming to cool the brain during the perfusion. The internal carotids were identified superior to the carotid bifurcation as the more posterior of the two major arteries (the more anterior vessel being the external carotid). After a brief dissection with scalpel and forceps to isolate approximately 2 cm of the vessel from surrounding tissue, 10G or 12G dispensing needles, used as cannulae, were inserted into the internal carotid arteries and secured via zip ties (**Table 1**). Once both carotid cannulae were in place, the perfusion was initiated. Occasionally one of the zip ties was not tied securely tightly enough, in which case the perfusion was momentarily stopped, and the cannulation of that artery was redone prior to restarting perfusion.

**Table 1:** Supplies and reagents for perfusion

Equipment / Chemical	Supplier	Product Identifier
Masterflex L/S economy drive	Cole-Parmer	7518-10
Masterflex easy-load pump head	Cole-Parmer	7518-10
Silicone tubing, 1/4" inner diameter	Novosci	SDB08
Straight barbed connector, 1/4" inner diameter	VWR	MFLX40614-43
Pump roller tubing, C-Flex	VWR	MFLX06424-24
Y-connector, 1/4" inner diameter	Cole-Parmer	MFLX30726-05
Male luer lock barb for 1/4" ID tubing	Qosina	11567
PendoTECH PressureMAT sensor monitor	BioPharm World	PMAT-S
Pressure monitor	PendoTECH	PMAT4A
Polycarbonate carboy	Nalgene	2322-0020PK
Neutral buffered formalin	Azer Scientific	NBF55G
Mannitol	Lab Alley	MANPU-1LB
Omnipaque™ 300 mgI/mL (iohexol)	Med-Plus	0407-1413-63
Green colored dye	Kroger	0001111002073
Dispensing needle, 10G or 12G	LabAider	B0B5D28CBM
Dispensing needle, 14G	BBTCTY	B07DZC225B
Zip tie	Have Me TD	B08TVLYB3Q
Zip tie gun	RV Rhodes	B0938PVFM9
Plastic tubing clamp	Bel-Art	F18228-0000
Plastic syringe, 140 ml	Cardinal Health	8881114055
Plastic syringe, 200 ml	A AKRAF	B0CJJDQHPH

Once the perfusion through the carotid arteries had begun, the bilateral vertebral arteries were usually clamped with hemostats to prevent outflow through them. However, in cases where no or only minimal flow was visualized returning through the vertebral arteries, the latter were not clamped, as this was not deemed necessary. Occasionally the external carotid arteries were also found to have flow of perfusate through them, most likely due to collateral circulation pathways. In that case, the leaking external carotid arteries were clamped with hemostats. In a small minority of cases (n = 5), the vertebral arteries were also cannulated and perfused through. In these cases, the vertebral arteries were cannulated with 14G dispensing needles functioning as catheters. The same perfusate solution and a separate perfusion circuit with the same design were used for the vertebral artery perfusion.

**Figure 1.**
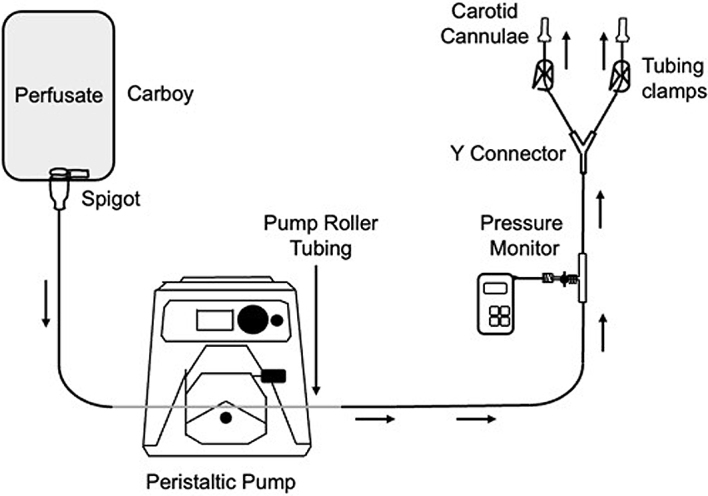
Diagram of one version of the perfusion circuit used.

Perfusion pressure was applied using a peristaltic pump system or by manual syringe injection. For pump-driven perfusion, we used an open circuit setup (**Figure 1**). For the peristaltic pump, we primarily used the Masterflex L/S Economy Drive (Cole-Parmer, Model #7554-80) with the Masterflex Easy-Load Pump Head (Cole-Parmer, 7518-10). For syringe-based perfusion, we manually injected fixative into the internal carotid arteries in a controlled, slow manner. We used multiple syringes, which were sized 140 ml, 200 ml, or both, and which were pre-filled with perfusate prior to the procedure.

Batches of perfusate were made in polycarbonate carboys (Thermo Scientific, 2322-0020PK). The base solution was 10 % neutral buffered formalin (NBF; Azer Scientific, NBF55G). In some cases, we added mannitol (Lab Alley, MANPU-1LB), primarily at a concentration of 10 % (v/v), although in a minority of cases 20 % (v/v) mannitol was used. For contrast-enhanced CT imaging studies, we used iohexol (Omnipaque™) at a concentration of 3 mgI/mL, although in a small minority of cases a concentration of 6 mgI/mL was used. We also added colored dye, typically green, to be able to better visualize the color changes of brain tissue on gross examination. Although in initial experiments the carboys were sometimes stored at room temperature prior to use, we later switched to storing the carboys at 4 °C until just before use, so that the solution was cold during perfusion. This was done to aid in more rapidly cooling the brain, and because we expected that cold perfusate might help to decrease tissue edema during perfusion.

For the cases with peristaltic pump-driven perfusion, which provides a constant flow, pressure was monitored using an inline pressure sensor previously connected to the perfusion circuit. Prior to cannulation, the circuit was primed by rapidly distributing fluid through all of the tubing to completely remove of visible air bubbles. We typically began with flow rates of approximately 16 ml/min (corresponding to a speed of 0.5 on the pump, with negligible differences when using a cannula size of 10G or 12G), and gradually increased the flow rate to 38 ml/min (speed of 1), 94 ml/min (speed of 2), or 147 ml/min (speed of 3), provided that the pressure was not too high and no spinal cord swelling or herniation were observed. It should be noted that these flow rates are relative to operating conditions such as the state of the tubing, air bubbles, or temperature. In initial experiments, the target perfusion pressure was between 50 to 100 mm Hg (0.97 to 1.93 PSI), although this was a loose guide. The primary factors used for adjusting the speed and deciding whether to stop perfusion were (a) the absence of rising pressure over time, which would indicate progressive resistance to perfusion and potential edema, (b) the absence of visible spinal cord bulging or herniation, and (c) complete clearance of blood from the draining effluent. Occasionally, especially in earlier cases, the pressure sensor malfunctioned - most likely due to water contamination introduced during cleaning – leading to erroneously high-pressure readings. When such malfunction was suspected, the recorded pressure data were excluded from the analysis.

Venous return during perfusion was monitored through the internal jugular veins (IJVs) and within the spinal canal. In most cases, a gradual transition in the color of return fluid from dark red to clear was observed. However, we noticed that this rapid clearing of the return fluid could also occur when perfusion was limited to a small area of the brain, rather than being widespread. Therefore, from a venous return perspective, the best sign for widespread perfusion across the brain was a slow, progressive transition of the return fluid from blood-colored to clear over the course of the procedure.

In addition to the variations in the approach described above, we also tried (a) different cannula sizes for the internal carotid arteries (10G or 12G) and (b) alternating perfusate delivery through a single carotid at a time by temporarily clamping the contralateral line. Qualitatively, neither of variation produced substantial differences in perfusion outcome, and hence were not considered in subsequent analyses.

### CT images

We aimed to perform neuroimaging both before and after perfusion, with the brain remaining inside of the skull. The CT scanner we used was the OmniTom® Elite (Neurologica, Danvers, MA), a 16-slice scanner. Images were viewed with the Osimis Web Viewer. Occasionally, one or both of these CT scans was not performed, for example if the CT scanner was not working at the time. For each of eight major vascular territories – corresponding to the territory supplied by the left and right anterior cerebral artery (ACA), middle cerebral artery (MCA), and posterior cerebral artery (PCA), as well as the cerebellum, we graded the extent of contrast visualized on the post-perfusion CT scans in a given territory on a 0–3 scale, with 0 indicating < 5 % extent of perfusion, 1 indicating 5–50 %, 2 indicating 50–95 %, and 3 indicating > 95 %. In some cases, the grades could not be completed for some of the areas in the brain because the available CT scan contained only part of the brain, not the whole brain. The extent of air bubbles in the CT scan across the brain was also graded on a 0–3 scale, with 0 indicating no apparent air bubbles, 1 minimal, 2 moderate, and 3 substantial. This grading was performed on all of the post-perfusion CT scans available via the collaboration of two graders. For a brain-wide assessment measure, we generated a separate score based on the sum of the grades across all evaluated areas, excluding cases with missing data from any vascular territory.

### Brain extraction procedure

Following perfusion, we performed brain extraction following standard autopsy procedures (Adams and Murray, 1982), with slight modifications aimed at minimizing tissue damage during removal. We used an oscillating saw to create a circumferential skull cut extending below the occipital protuberance to have better access to the posterior cranial fossa (Felle et al., 1995; Hlavac et al., 2018). In addition, we performed a median sagittal cut to aid in decreasing trauma during removal (Robertson et al., 2024). After carefully cracking the inner table using an osteotome and mallet, we removed the calvaria, incised the dura along the saw lines, and transected the falx cerebri and tentorium. The brain was then gently extracted by sequentially transecting the connecting cranial nerves and arteries. When the brain was well perfused, it became firmer, which appeared to aid in mitigating damage during removal.

### Gross examination

Following extraction, digital photographs of the brain were taken from multiple angles prior to immersion fixation, to avoid fixation-induced alterations in tissue color. After the photographs were taken, the brains were completely immersed in a plastic container with 10 % neutral buffered formalin (NBF), and stored at 4 °C (McKee, 1999). The gross examination images were graded analogously to the CT images, evaluating the territories supplied each the left and right ACA, MCA, and PCA, as well as the cerebellum. The features used for estimating the extent of perfusion on the images were (a) clearance of blood from surface blood vessels, (b) apparent stiffness of tissue, and (c) color change of tissue, from pink in the case of no perfusion to pale or pale/colored dye-tinged in the case of well-perfused tissue. In some cases, specific brain areas could not be graded due to incomplete visualization in the available images. As with the CT scans, two independent raters evaluated the same subset of data. For a brain-wide assessment measure, a separate score was created based on the sum of the grades across all of the graded areas, removing cases with missing data from any region.

### Histology

In a subset of 11 cases, we sectioned the brains and dissected approximately 1x1x1 cm samples for histology. Specifically, we collected grey matter samples from the left and right cerebellum, frontal cortex, occipital cortex, temporal cortex, thalamus, and white matter samples from the left and right temporal cortex, yielding a total of 12 distinct brain regions. As a control, one additional brain (from an 83-year-old donor with a PMI of 27 hours) that was immersion-fixed rather than perfusion-fixed, was processed to obtain samples from the same 12 brain regions. Brain tissue sampled for light microscopy was placed into cassettes for processing and embedded in paraffin. Paraffin-embedded brain sections 6 μm thick were baked, deparaffinized, and stained for Hematoxylin and Eosin (H&E). Digital images of the stained sections were captured at 40X as whole slide images (WSIs) using the Aperio GT450 high-resolution scanner (Leica Biosystems). To analyze the data, we graded the extent of vessel clearance across each of the WSIs on a 0–3 scale, with 0 indicating < 5 % clearance of blood vessels, 1 indicating 5–50 %, 2 indicating 50–95 %, and 3 indicating > 95 %. Vessel clearance refers to the absence of intravascular material from both small and large vessels, as in some cases there was clearance from the large vessels but not the small vessels.

As an additional brain banking cohort for a comparison to immersion fixation, we also graded the vessel clearance in the frontal cortex from brain hemisphere specimens that were immersion fixed with 10 % neutral buffered formalin, stained for H&E, and captured as WSIs (Garrood et al., 2025). We graded these WSIs using the same 0–3 vessel clearance scale as above. The grades from the perfusion fixed samples and the immersion fixed samples in this separate cohort from the frontal cortex (the only region available for comparison in both cohorts) were then compared with a t-test.

### Interrater reliability scores

For both the gross examination images and CT scans, on a subset of the data, perfusion quality grades were performed independently by two raters. For the CT scans, this subset included the air bubble assessment as well. For all of the histology WSIs, perfusion quality grades were performed independently by the two raters. The interrater reliability for each of these types of grades was then calculated using the intraclass correlation coefficient (ICC), applying a model with agreement estimation, single unit of analysis, and two-way random-effects. The ICC values were interpreted using previously established guidelines (Koo and Li, 2016). If instances of discrepancies between the raters, the final grade was determined via a consensus review between the two raters.

## Results

### Approach to brain perfusion

In preliminary experiments, we cannulated and perfused the common carotid arteries using an anterior cervical approach that has previously been demonstrated to provide high-quality perfusion of the brain (Insausti et al., 2023). However, in our experience, especially in donors with PMIs longer than 24 hours, this approach sometimes resulted in substantial perfusion of facial tissues, causing facial edema but minimal or no perfusion of the brain. Additionally, when using the anterior cervical approach for carotid cannulation, backflow was observed from the caudal severed ends of the carotid arteries. This was attributed to perfusion through the circle of Willis, and subsequent backflow into the systemic circulation through the vertebral arteries, and out through the low-pressure system created by the open carotid arteries. Therefore, instead of the fixative perfusing the brain parenchyma and returning through the intended venous drainage pathways, the vertebral arteries created a shunt that diverted perfusate away from this route to the systemic circulation.

In order to more quickly access the internal carotid arteries and prevent the problem of vertebral artery shunting, we switched to performing perfusion on the isolated cephalon, a method previously used in neuroanatomical studies and neurosurgical training (Sanan et al., 1999; Alvernia et al., 2010; Benet et al., 2014; Turkoglu et al., 2014; Mignucci-Jiménez et al., 2024). This approach facilitates rapid visualization and clamping of the vertebral arteries, effectively preventing perfusate from shunting through this pathway. Even with this more focused approach, we sometimes observed perfusion to the superior facial tissues, particularly in the periorbital region. This phenomenon likely reflects collateral circulation via the ophthalmic artery, as previously described (Kalimo et al., 1974). The extent of periorbital perfusion appeared to be inversely related to the degree of cerebral parenchymal perfusion, although in most cases, periorbital distribution of perfusate remained minimal. With cannulation of the internal carotid arteries and clamping of the vertebral arteries, the majority of the perfused fluid appeared to flow through the brain vasculature. Using this approach, total perfusion volumes ranged from 0.1 to 2.8 liters per case (mean: 1.16 liters), delivered over 4 to 45 minutes (mean: 19.9 minutes).

### Spinal canal fluid return

During perfusion with the isolated cephalon approach, we observed substantial fluid outflow through the open spinal canal in most cases. This occurred rapidly, often within a few seconds after the initiation of mechanical perfusion, and sometimes before fluid returned from any other site. Moreover, outflow through the spinal canal often represented substantial source of perfusate return, often exceeding the volume returning through the IJVs. Consistent with perfusate entering the CSF compartments, some degree of contrast was frequently visible at least in the ventricles on post-perfusion CT scans. Importantly, superficial cerebral veins were often cleared even in cases where nearly all fluid outflow occurred through the spinal canal. Additionally, when the cephalon was repositioned during the procedure, outflow from the IJVs occasionally ceased completely, possibly due to constriction. When the IJV flow stopped, spinal canal outflow appeared to increase proportionally, without any apparent change in the measured perfusion pressure or the color of the returning fluid. This shift in relative flow through the spinal canal was reversible upon further repositioning of the cephalon. These observations suggest functional communication between the venous and CSF compartments within the intracranial cavity. One possible explanation for this phenomenon is retrograde flow through arachnoid villi, which has been suggested to allow communication between venous and CSF compartments under certain conditions, including postmortem (Potts et al., 1972; Alvernia et al., 2010; Proulx, 2021). Another possible route involves the perivascular spaces surrounding cerebral veins, which have been found to communicate with the CSF compartment postmortem (Ma et al., 2019). Regardless of the exact mechanism, which remains unclear, these findings indicate that spinal canal outflow can effectively substitute for venous outflow, and is therefore not necessarily indicative of perfusion failure.

In some cases, we also observed sudden displacement of the spinal cord or adjacent nervous tissue during perfusion. This finding is indicative of brain herniation, which in turn reflects edema in at least some regions of the brain (Alvernia et al., 2010). Herniation occurred in 14 out of the 66 cases (21.2 %) using the isolated cephalon approach for which the presence or absence of this outcome was recorded. Qualitatively, it appeared to be more likely in cases with longer PMIs, but this was not a statistically significant effect in our sample (average PMI of cases with herniation = 53.4 hours, average PMI of cases without herniation = 32.5 hours, t-test p-value = 0.11). Herniation often occurred shortly after an increase in perfusion flow rate. The mechanism by which postmortem perfusion may cause edema in some cases is not clear. We propose that it most likely involves increased BBB permeability, which is known to increase progressively due to ischemia during the postmortem interval (Krassner et al., 2023). When BBB integrity is compromised, perfusate may extravasate into the parenchyma more readily than it circulates through the vascular system. This process could theoretically affect different brain regions heterogeneously, depending on variations in baseline BBB integrity and the rate of BBB degradation. Some protocols designed for vascular visualization have suggested occluding the spinal canal with bone wax during isolated cephalon perfusion to prevent fluid loss through this route (Sanan et al., 1999). However, we intentionally maintained an open spinal canal as we expected that maintaining an outlet for fluid to leave the cranial cavity may help to decrease edema. Additionally, this configuration allowed the observation of spinal cord bulging as a proxy for increased intracranial pressure, which we consider a useful marker for when perfusion should be slowed or terminated to prevent further parenchymal damage from edema and associated herniation (Alvernia et al., 2010).

### Quality of perfusion in gross images

**Figure 2.**
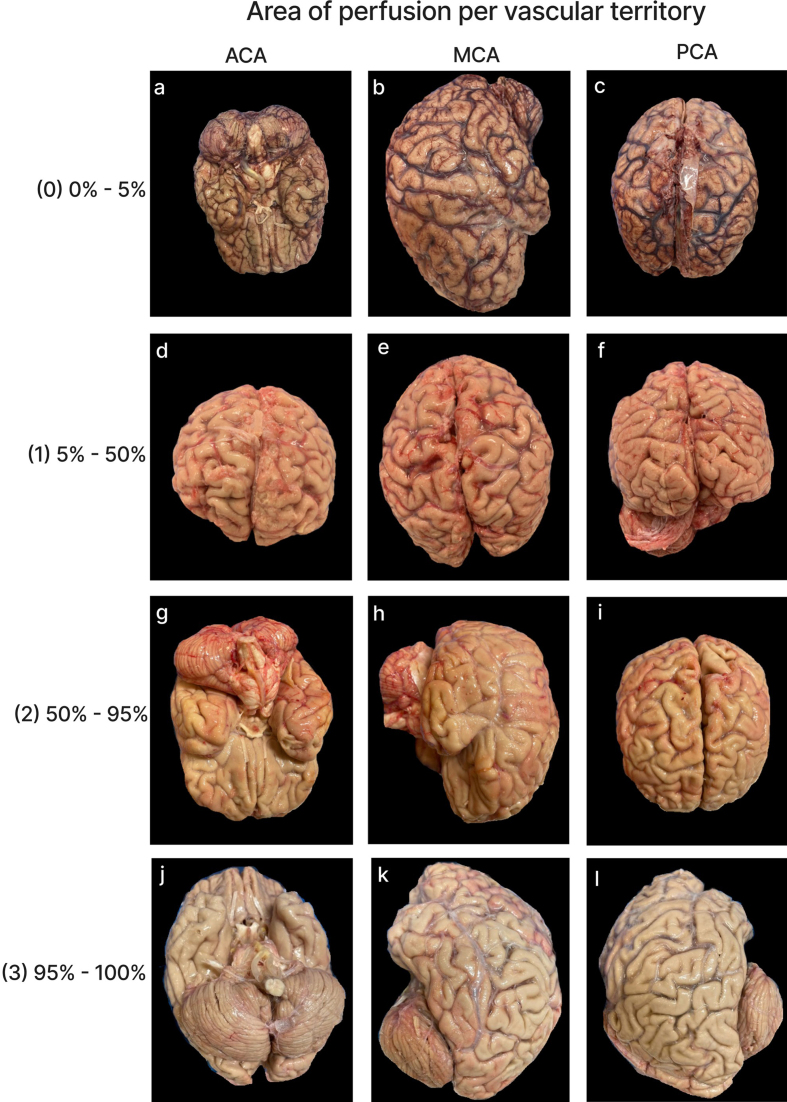
Representative gross examination images showing perfusion rated to be each of the four grades (0, 1, 2, and 3) with in the vascular distributions of the anterior cerebral artery (ACA), middle cerebral artery (MCA), and posterior cerebral artery (PCA). For grade 0, we did not have a single brain with a grade of 0 in all three regions, so different brain samples were used for each representative image. Donor IDs: 169 (**a**), 183 (**b**, **c**), 205 (**d**, **e**, **f**), 197 (**g**, **h**, **i**), 126 (**j**, **k**,** l**).

We developed a semi-quantitative rating scale to measure the extent of tissue perfusion in areas corresponding to each of six major cerebral arteries (i.e. the left and right anterior cerebral artery, middle cerebral artery, and posterior cerebral artery), as well as both sides of the cerebellum. Perfusion quality was assessed based on degree of pallor, clearance of surface blood vessels, visible tissue stiffness, and the presence of colored dye when used at a concentration sufficient for visualization (**Figure 2**). For a subset of data, perfusion quality based on these images was graded separately by two independent raters. These grades had an ICC of 0.691 (95 % CI 0.542–0.799), indicating good interrater reliability. Qualitatively, we found that in nearly all cases, perfusion appeared patchy achieved across the surface of the brain – both between and within vascular territories, for reasons that remain unclear.

**Figure 3.**
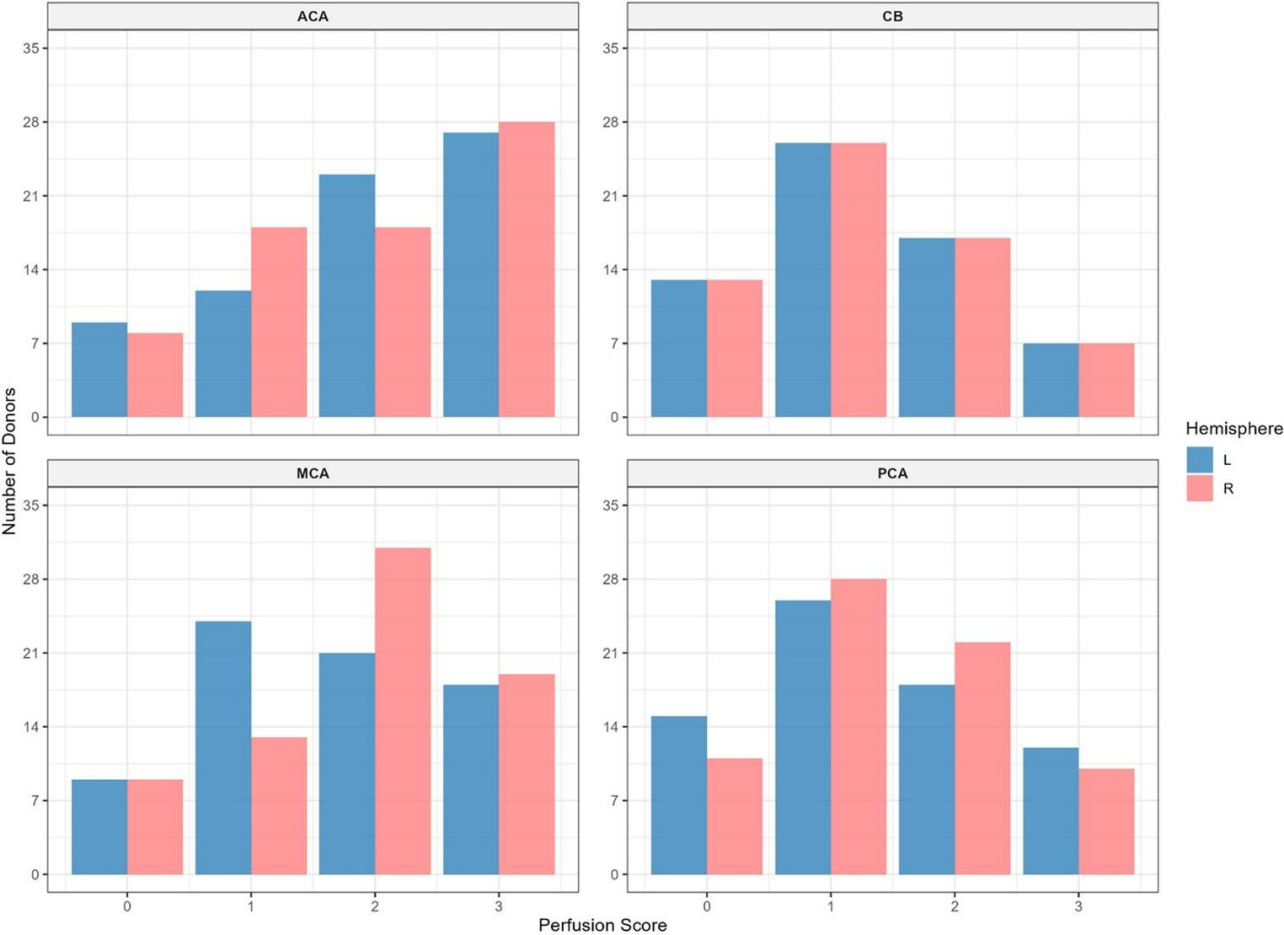
Histogram of the perfusion quality grades based on gross examination. Quality is graded on a 0–3 scale, where 0 indicates minimal perfusion and 3 indicates maximal perfusion in each region. Red bars represent right hemisphere; blue bars represent the left hemisphere. ACA: Anterior cerebral artery; MCA: Middle cerebral artery; PCA: Posterior cerebral artery; CB: Cerebellum.

The mean perfusion quality grades across both hemispheres were 1.94 ± 0.09 (standard error of the mean) for the ACA distribution, 1.75 ± 0.08 for the MCA, 1.75 ± 0.08 for the PCA, and 1.29 ± 0.08 for the cerebellum (**Figure 3**). There was no significant difference between the grades in the ACA and MCA distributions (t-test, p-value = 0.11). The grades in the ACA and MCA were each significantly higher than those in the PCA (t-test, p-values = 1.2e-5 and 0.003, respectively) and the cerebellum (t-test, p-values = 1.1e-7 and 7.7e-5, respectively). This indicates that perfusion quality was relatively higher in areas supplied by the anterior circulation (ACA and MCA) compared to those supplied by the posterior circulation (PCA and cerebellum). On the other hand, no consistent difference was found in perfusion quality between the left and right hemispheres, although some brains exhibited a slightly greater perfusion on one side than the other.

To further analyze differences in perfusion between the anterior and posterior circulation, we focused on the cases for which perfusion of the isolated cephalon was performed solely through the internal carotids, which was the majority of total cases (62/77). In such cases, the primary way that perfusate can reach the posterior circulation is via the circle of Willis, which may however be absent, stenotic, or have perfusion impairment due to agonal or postmortem factors. After the initiation of mechanical perfusion through the carotid arteries, the degree of flow through the circle of Willis can be ascertained based on the amount of fluid that returns through the vertebral arteries, which we initially left patent. Although we recorded the presence or absence of flow in a subset of cases, we more commonly recorded whether we clamped the vertebral arteries, which we only did when a sufficient amount of flow was going through them. As a result, clamping of the vertebral arteries can serve as a proxy for the presence of flow through the circle of Willis in a given case.

We created a measure of the divergence between the perfusion quality in the anterior and posterior circulations, which was the difference of the sums of the grades in the areas supplied by the ACA and MCA minus the sums of the grades in the area supplied by the PCA and the cerebellum. We measured the average difference in the anterior-posterior divergence in perfusion quality between the cases in which at least one of the vertebral arteries were clamped (46/59) and cases in which none were clamped (13/59), and found no significant difference (t-test, p-value = 0.32). These results suggest that, when fluid is perfused through the internal carotids, the observed flow through the vertebral arteries is not a significant predictor of divergence in the perfusion quality between the anterior and posterior circulations of the brain, at least in this sample.

### Quality of perfusion in CT images

**Figure 4.**
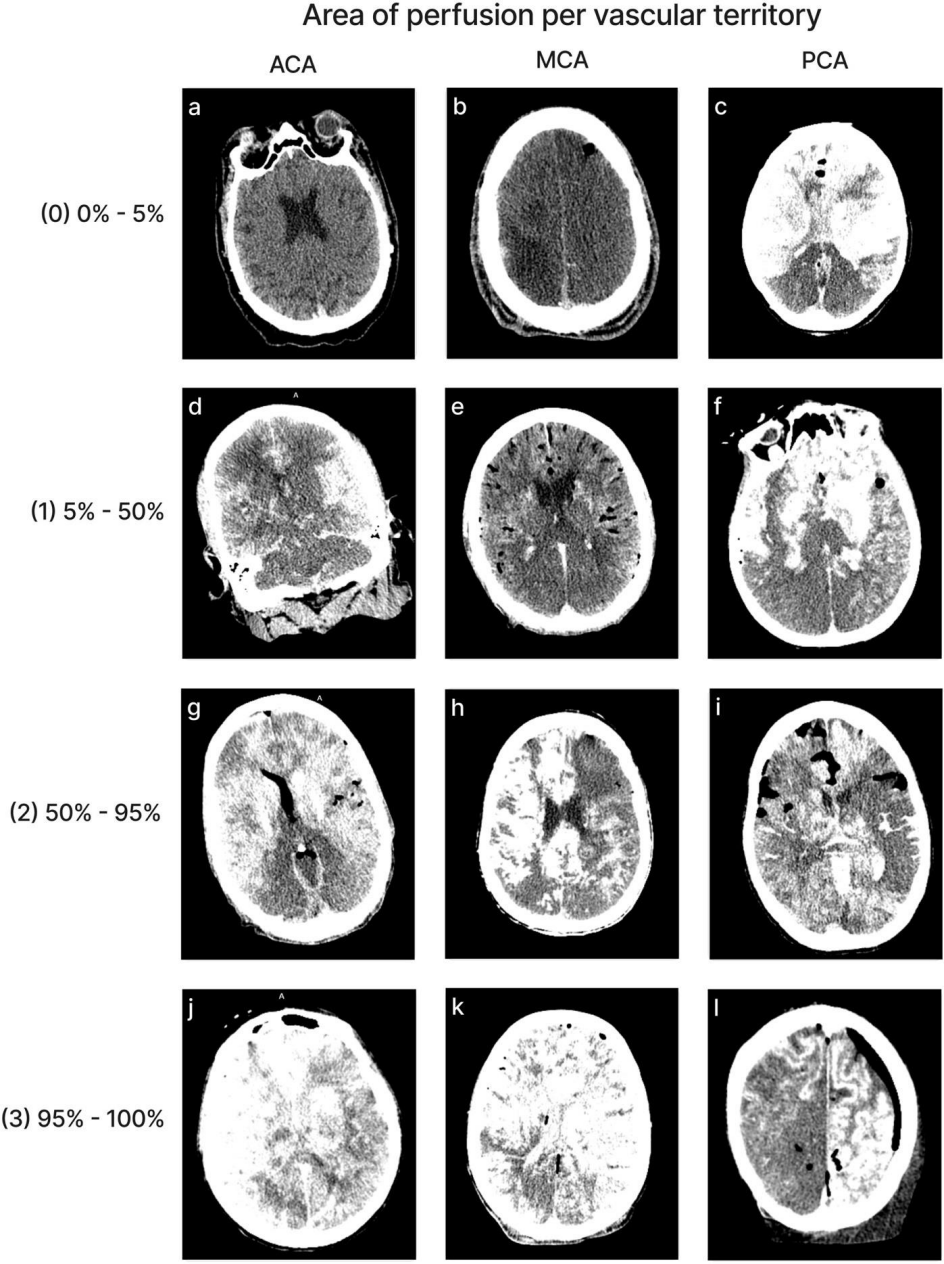
Representative images of CT scans that demonstrate the grading schema. All images show the grading for both the left and right sides of the respective region, except for image(**l**), in which only the left side represents the correct grading. Images follow the standard radiological convention, with the right side of the image corresponding to the donor’s left side and vice versa. Donor IDs: 136 (**a**), 71 (**b**), 185 (**c**), 179 (**d**), 197 (**e**), 201 (**f**), 195 (**g**), 5 (**h**), 84 (**i**), 203 (**j**), 206 (**k**), 142 (**l**).

**Figure 5.**
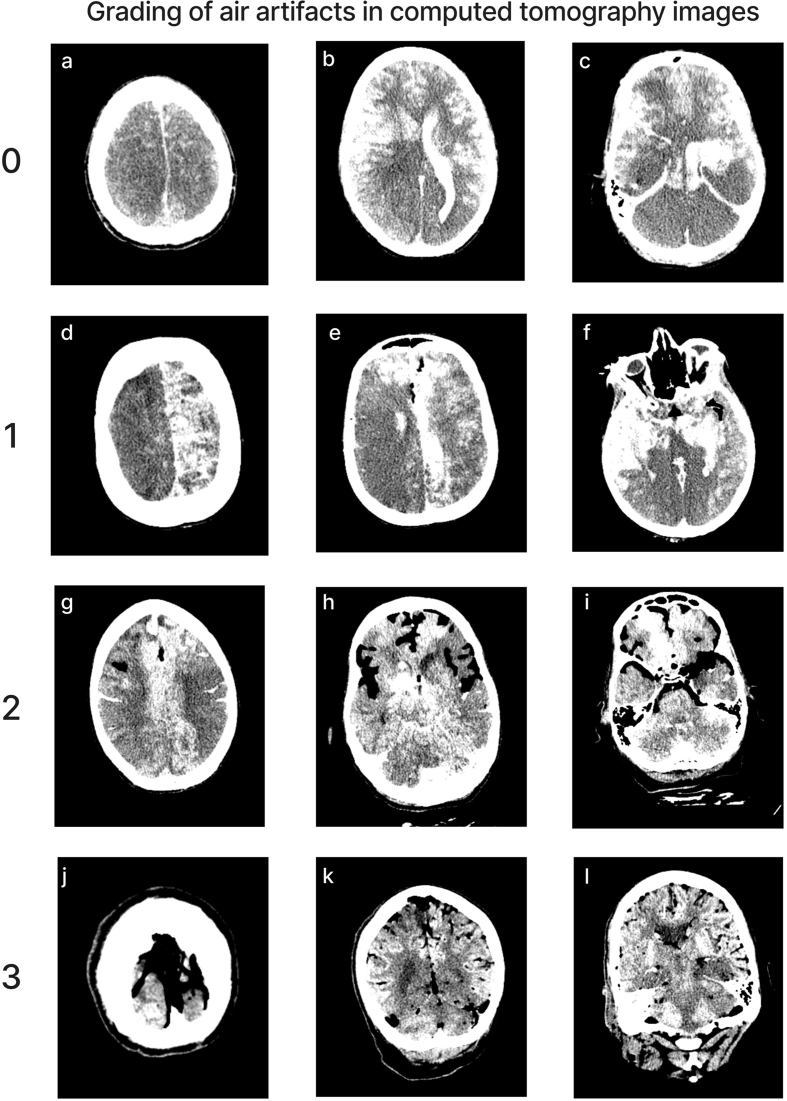
Representative images of CT scans showing the grading of air bubble extent across the brain. Donor IDs: 202 (**a**, **b**, **c**), 207 (**d**, **e**, **f**), 184 (**g**, **h**, **i**), 160 (**j**, **k**, **l**).

Perfusion quality based on CT scans was graded on a semi-quantitative scale, reflecting the estimated percentage of contrast agent present in different brain regions (**Figure 4**). We also developed a semi-quantitative grading scale to assess the extent of air bubbles observed in some CT scans (**Figure 5**). For a subset of cases, the images were graded separately by two independent raters, yielding an ICC of 0.536 (95 % CI: 0.293–0.705), indicating fair interrater reliability. Consistent with the observations from gross examination images, the CT scans revealed patchy distribution of contrast in nearly all cases, both across and within vascular territories.

**Figure 6.**
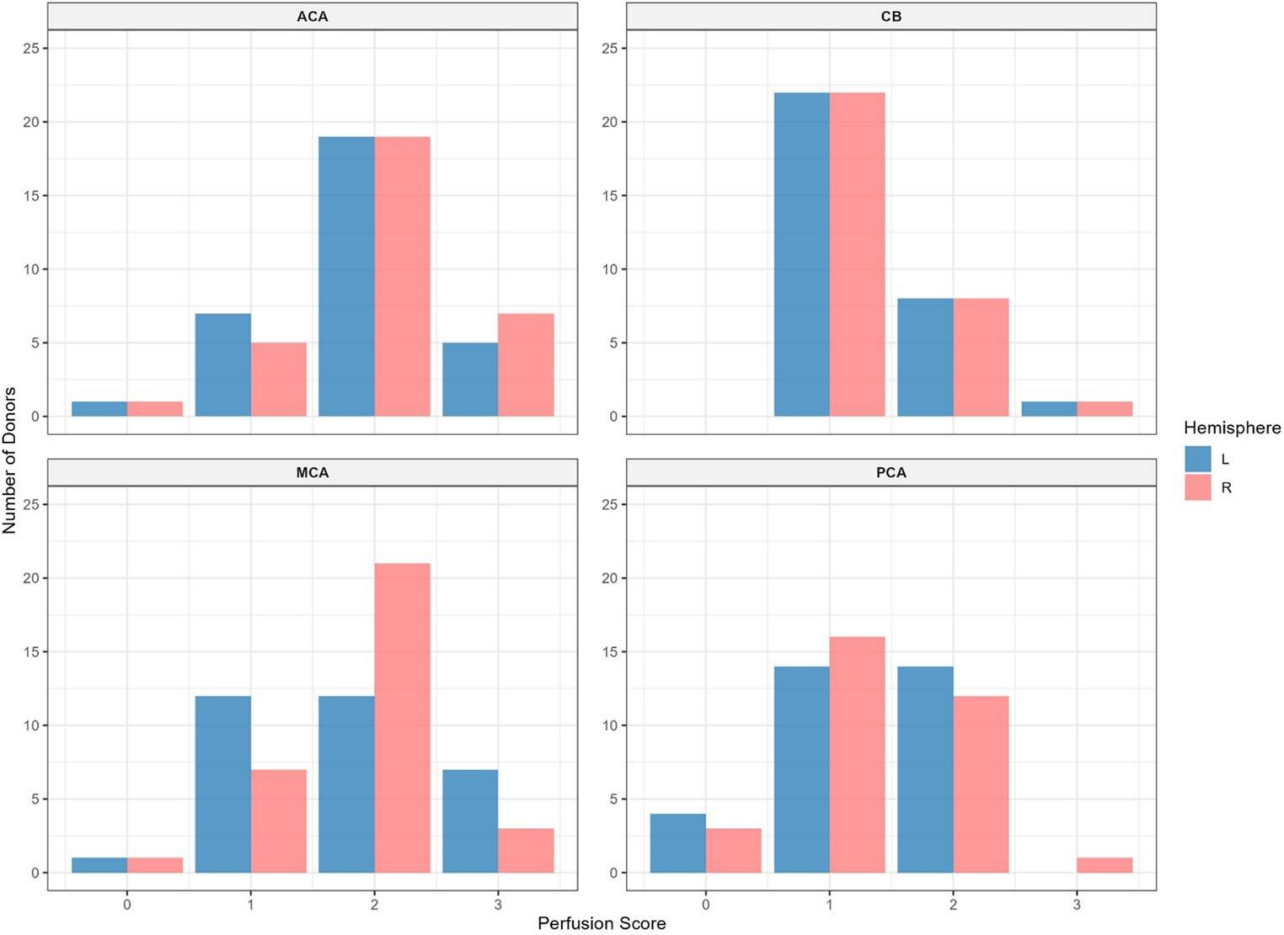
Histogram of perfusion quality grades based on CT images. Perfusion quality is graded on a 0–3 scale, where 0 indicates minimal perfusion and 3 indicates maximal perfusion in each region. Red bars represent the right hemisphere; blue bars represent the left hemisphere. ACA: Anterior cerebral artery; MCA: Middle cerebral artery; PCA: Posterior cerebral artery; CB: Cerebellum.

The mean perfusion quality grades based on CT scans across both hemispheres were 1.94 ± 0.09 for the ACA distribution, 1.80 ± 0.09 for the MCA, 1.33 ± 0.09 for the PCA, and 1.32 ± 0.07 for the cerebellum (**Figure 6**). There was no significant difference between the grades in the ACA and MCA distribution (t-test p-value = 0.27). The grades in the ACA and MCA were each significantly higher than those in the PCA (t-test, p-values = 2.6e-6 and 0.0003, respectively) and the cerebellum (t-test, p-values = 2.3e-7 and 6.8e-5, respectively). Therefore, as with the gross examination data, average perfusion quality graded on CT scans was found to be higher in the regions supplied by the anterior circulation than in those supplied by the posterior circulation.

**Figure 7.**
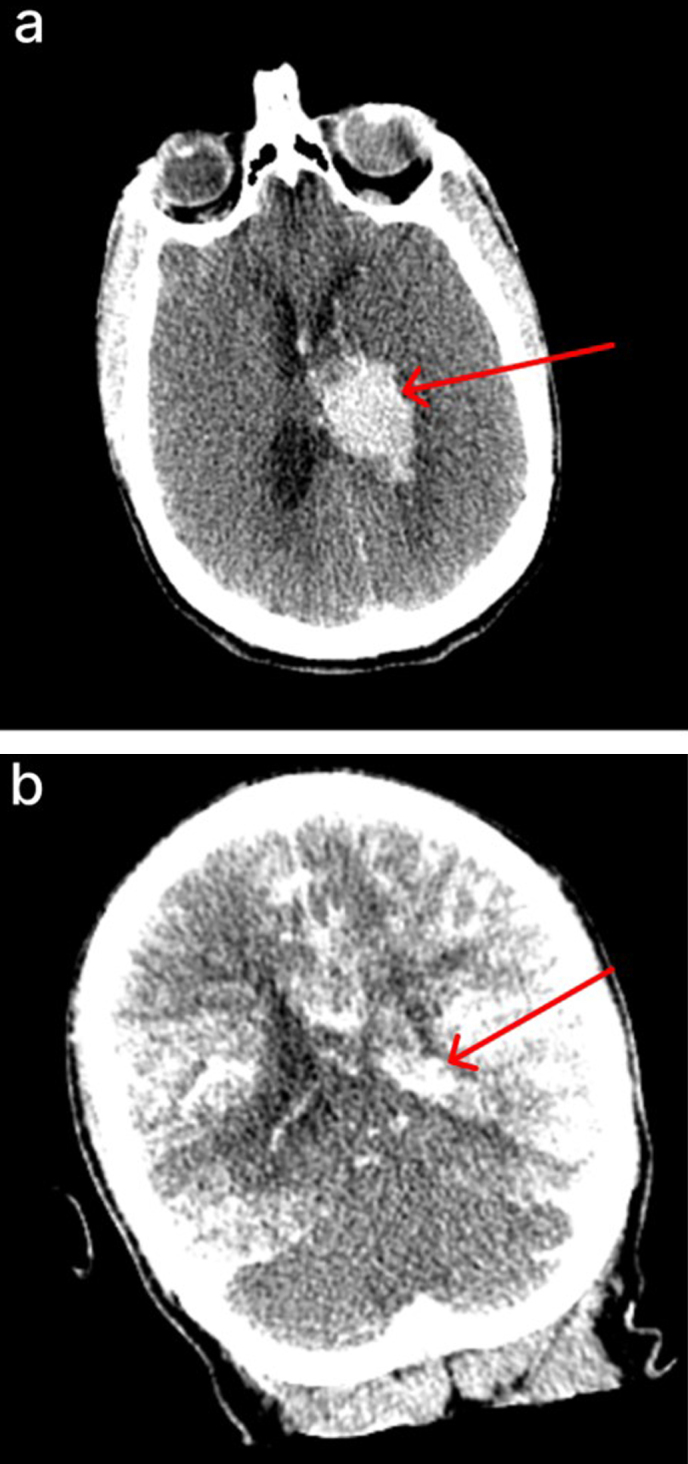
CT scans of a brain donor with observed herniation of tissue through the spinal canal during perfusion, without no clear evidence of significant anatomical sequelae. **a**: Pre-perfusion axial CT image of a donor with a known hemorrhagic stroke, showing a large intraventricular clot (red arrow). **b**: Post-perfusion coronal CT image showing partially successful perfusion, as evidenced by iohexol distribution, with persistent intraventricular clot (red arrow). During perfusion of this brain, a suspected herniation event occurred, as tissue abruptly emerged from the spinal canal, prompting immediate termination of the perfusion. However, there was no clear evidence of anatomical damage due to the herniation on the CT scan, as the ventricles remained of the same size and no midline shift was observed. Donor ID 179, PMI of 70.5 hrs.

In four of the cases in which herniation of tissue through the spinal canal was observed, we had CT scans of the brain following perfusion available, which we analyzed further. In one of these cases, there was evidence of a midline shift; however, this donor had a history of an arteriovenous malformation in the frontal lobe, and no pre-perfusion CT scan was available for comparison, so the arteriovenous malformation may have been present before the perfusion. In another case, the donor died of an aneurysm, which is visible on the CT scan, but there was no midline shift and the ventricles were of normal size. In another case, the brain appeared to have smaller-than-normal ventricles, but there was no midline shift, and there was no pre-perfusion CT scan available for comparison. Finally, in one of these cases, the donor died from a hemorrhagic stroke, and there was a large clot visible on the pre-perfusion CT scan, but after perfusion, there was no change in the ventricles or evidence of midline shift (**Figure 7**). Taken together, these results are difficult to interpret, because many of the cases in which herniation occurred had a history of brain pathology, which may be a predisposing risk factor for herniation. However, our data suggests that the herniation event does not necessarily lead to significant changes in the anatomical condition of the brain as visualized on CT.

### Quality of perfusion based on histology

**Figure 8.**
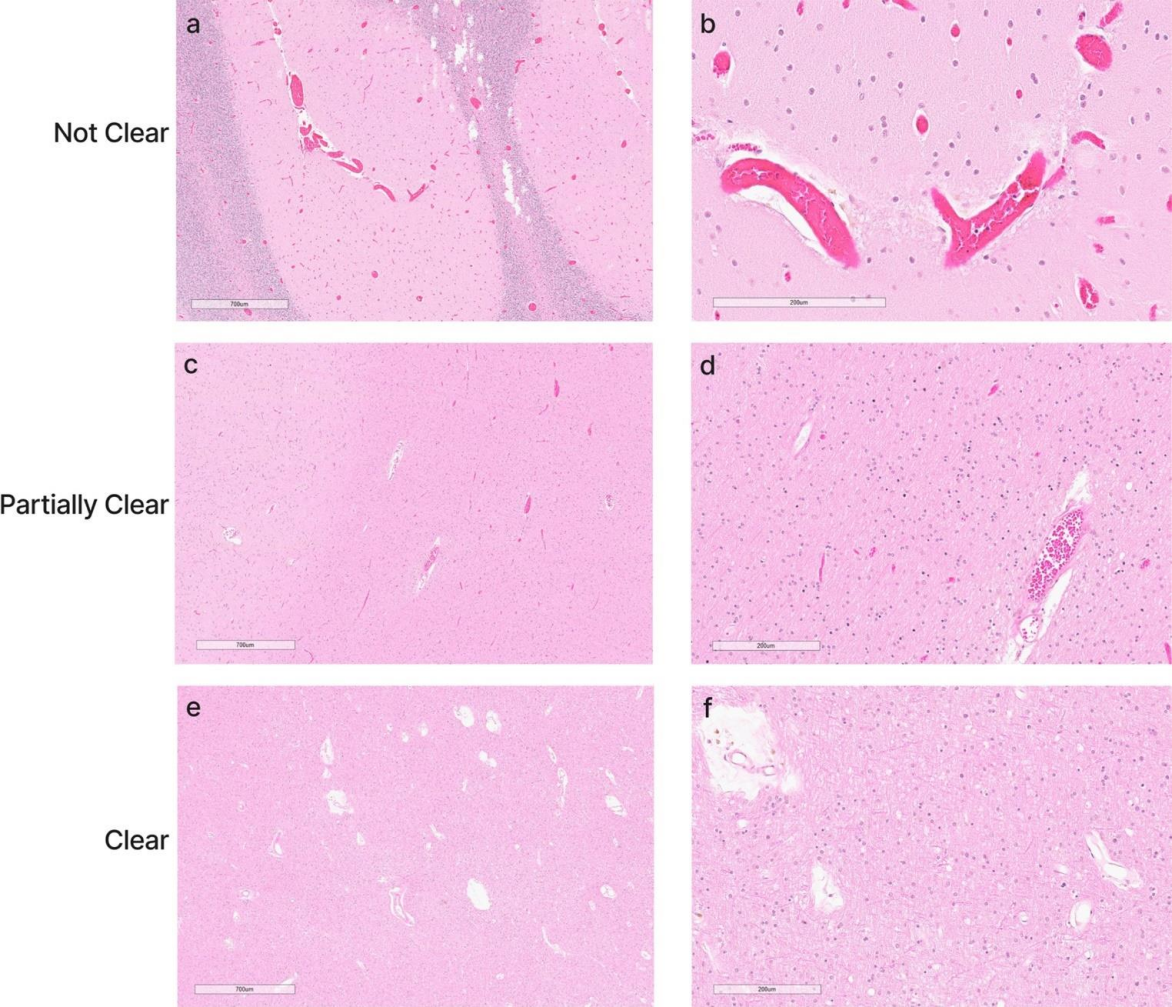
Representative histology images showing the degree of blood vessel clearance. This degree of clearance was determined across the entire WSI to determine the perfusion quality of each tissue sample. Donor IDs 147 (**a**, **b**), 78 (**c**, **d**), and 142 (**e**, **f**). Scale bars: 700 μm (**a**, **c**, **e**) and 200 μm (**b**, **d**, **f**). Scale bars: **a**, **c**, **e**: 700 μm; **b**, **d**, **f**: 200 μm.

**Figure 9.**
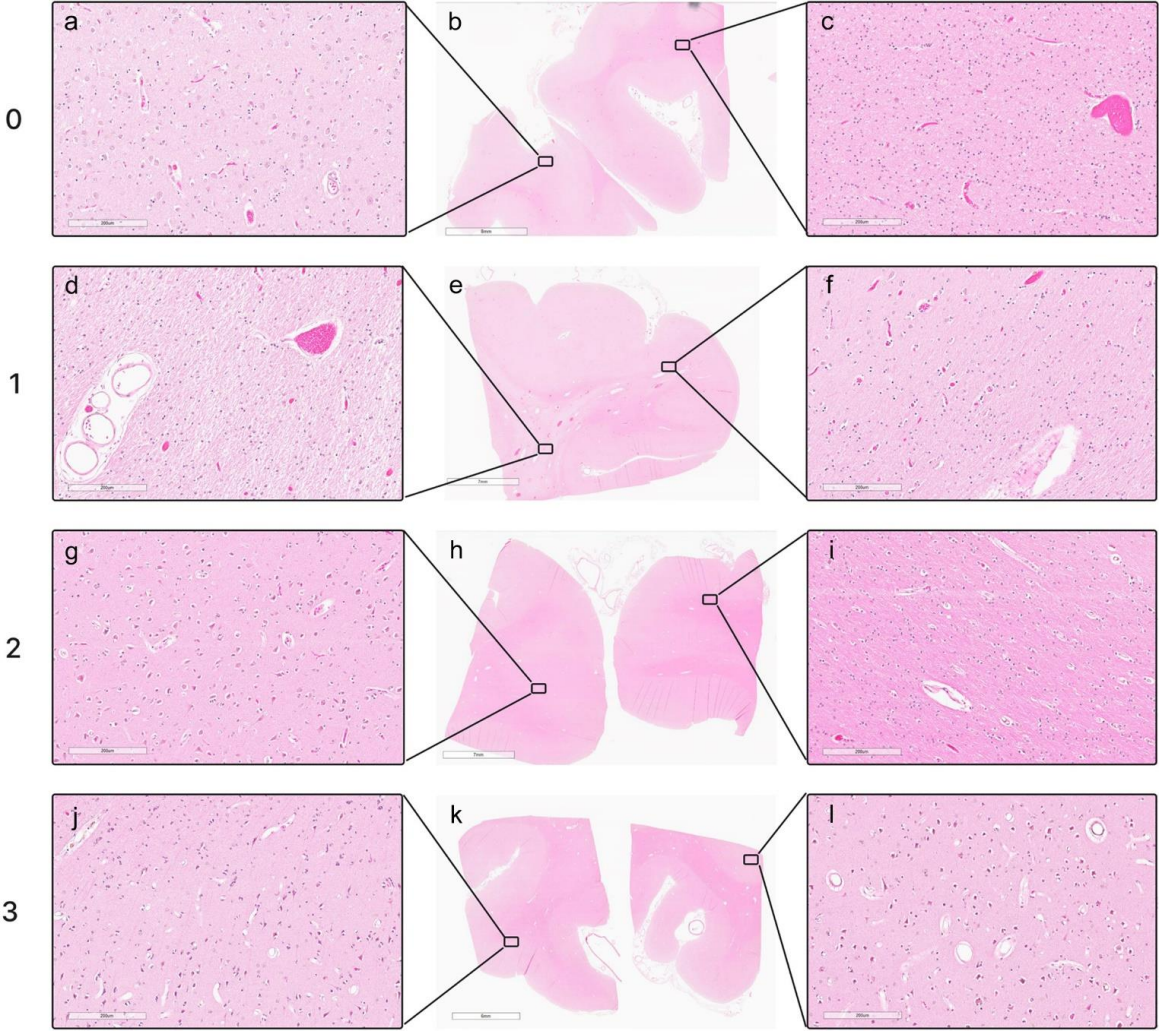
Representative histologic perfusion quality grades in WSIs. Grades were assigned on a 0–3 scale, where 0 indicates < 5 % clearance of intravascular material from vessels, 1 indicates 5–50 %, 2 indicates 50–95 %, and 3 indicates > 95 %. Grade 0: donor #166, left frontal cortex (**a**, **b**, **c**). Grade 1: donor #147, right occipital cortex (**d**, **e**, **f**). Grade 2: donor #107, right occipital cortex (**g**, **h**, **i**). Grade 3: donor #142, right occipital cortex (**j**, **k**, **l**). Scale bars: 200 μm (**a**, **c**, **d**, **f**, **g**, **i**, **j**, **l**), 8 mm (**b**), 7 mm (**e**, **h**), and 6 mm (**k**).

The quality of perfusion on histology was graded based on the clearance of intravascular material from blood vessels, with each vessel as not cleared, partially cleared, or fully cleared (**Figure 8**). The extent of clearance across all blood vessels in the WSI was then graded on a 0–3 scale to estimate the perfusion quality of that tissue sample (**Figure 9**). These grades were found to have an ICC of 0.804 (95 % CI 0.675–0.885), indicating excellent interrater reliability. As a control, we also performed histology on the brain of one donor (#166) that was exclusively preserved via immersion fixation, which yielded grades of 0 in six brain regions and grades of 1 in the remaining six brain regions. For additional comparison, we graded the vessel clearance in a larger set (n = 36) of exclusively immersion-fixed brain frontal cortex from a previously described cohort (Garrood et al., 2025). We found that vessel clearance was significantly higher in perfusion-fixed samples compared with immersion-fixed samples (mean perfusion-fixed sample grade = 1.59, mean immersion-fixed sample grade = 0.42, t-test, p-value = 1.56e-7). These results demonstrate that vessel clearance – and therefore our grading metric – is not entirely specific to perfusion, and that the partial clearance of intravascular material from blood vessels can also result from other mechanisms. However, vessel clearance is significantly greater in perfusion-fixed samples, consistent with the expected effect of perfusion in removing intravascular material.

**Figure 10.**
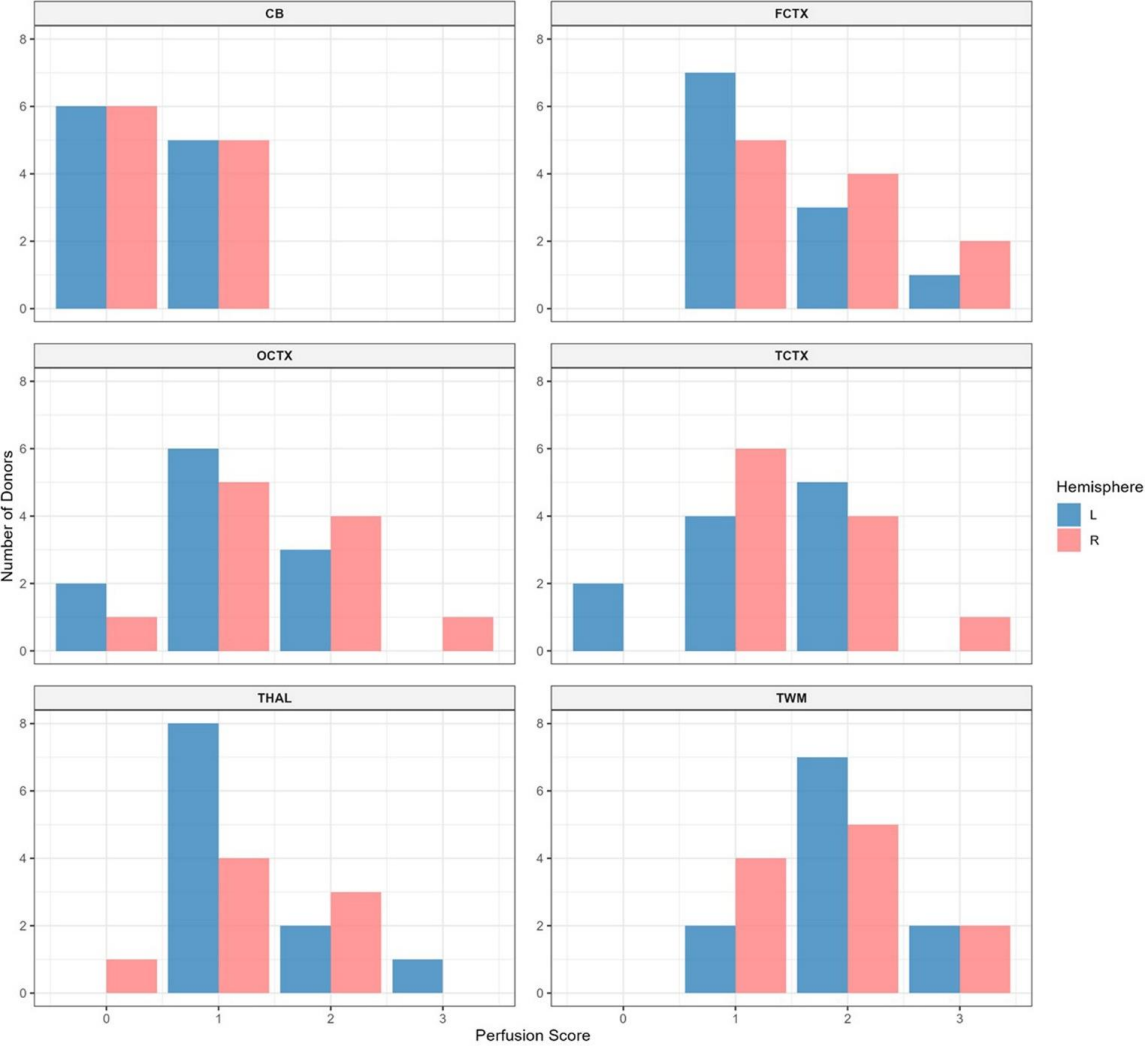
Histogram of histology perfusion quality grades. Higher grades correspond to a greater extent of vessel clearance across the whole slide image. Red bars represent the right hemisphere; blue bars represent the left hemisphere. CB: Cerebellum; FCTX: Frontal cortex; OCTX: Occipital cortex; TCTX: Temporal cortex; THAL: Thalamus; TWM: Temporal white matter.

The mean perfusion quality grades based on histology, averaged across both hemispheres, were 1.91 ± 0.15 for the temporal white matter, 1.59 ± 0.16 for the frontal cortex, 1.41 ± 0.16 for the temporal cortex, 1.32 ± 0.15 for the thalamus, 1.27 ± 0.15 for the occipital cortex, and 0.45 ± 0.11 for the cerebellum (**Figure 10**). The cerebellum exhibited significantly lower perfusion quality than the other regions, including the thalamus and occipital cortex (t-test, p-values = 6.4e-5 and 0.0002, respectively). The occipital cortex, in turn, had significantly lower average grades than the temporal white matter, but not the frontal cortex (t-test, p-values = 0.0059 and 0.167, respectively). These data suggest that average perfusion quality with our approach is relatively lower in the cerebellum, a region supplied by the posterior circulation.

In the histology data, we found that, frequently but not always, larger vessels were cleared of intravascular material while adjacent capillary networks predominantly remained largely filled with blood cells and other aggregated blood elements. This suggests that, even when large surface vessels appear cleared on gross examination and contrast is visible on CT imaging, portions of the capillary bed may remain largely unperfused. Notably, in such cases, it is unclear whether the subset of capillaries showing evidence of perfusate flow is sufficient for tissue perfusion, or whether arteriovenous shunts may allow significant portions of the perfusate to bypass the capillary beds entirely (Duvernoy et al., 1981; Grabherr et al., 2008).

### Correspondence between the quality metrics

**Figure 11.**
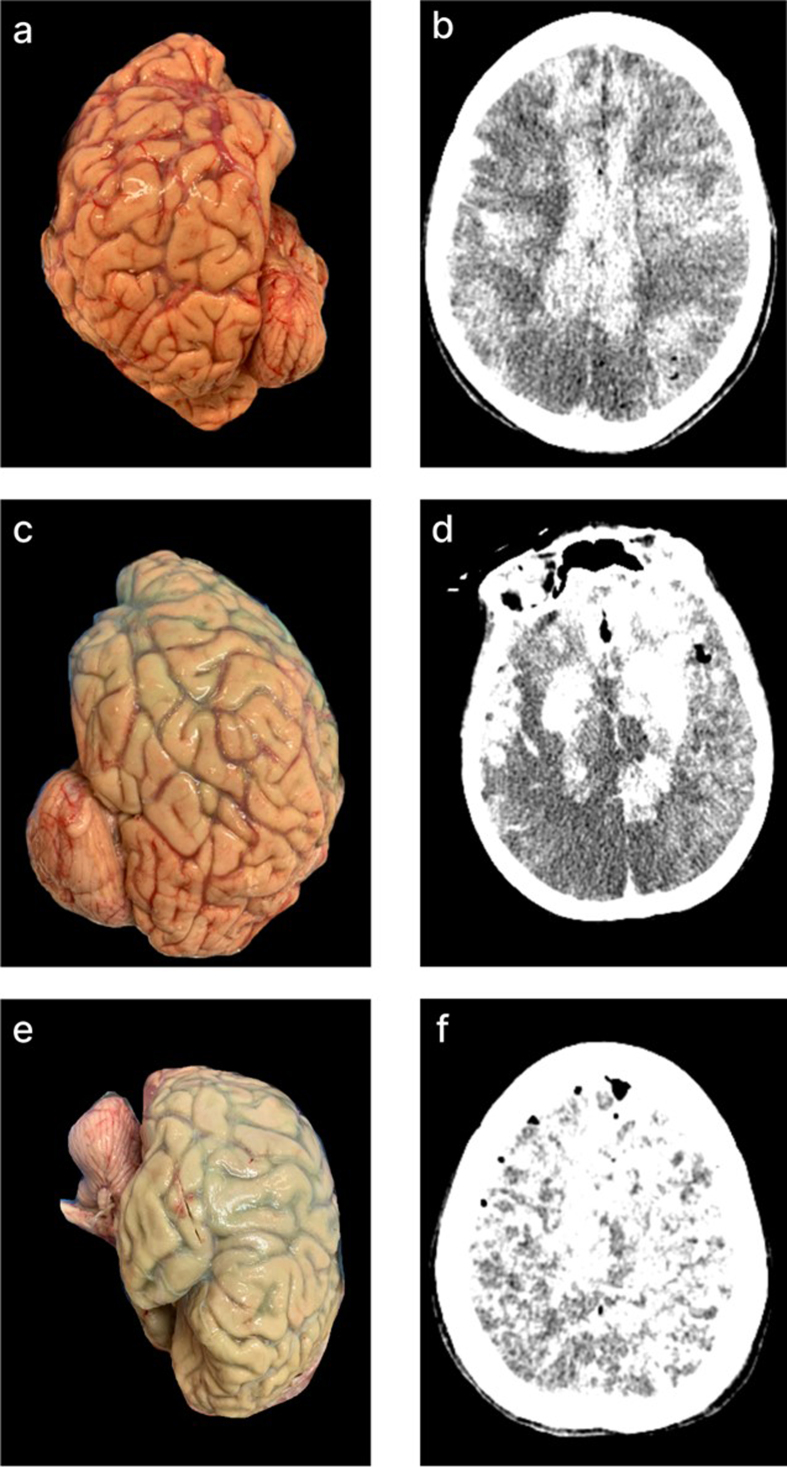
Representative matched gross examination and CT images illustrating spatial correlations in perfusion quality across modalities. On CT images (**b**, **d**, **f**), following standard radiologic convention, the right side of the image corresponds to the left side of the brain. In one of these perfused brains, a clear anterior-to-posterior gradient of perfusion is observed, with relatively better perfusion in the anterior regions and relatively worse perfusion in the posterior regions; this gradient is visible in both gross examination images (**c**) and CT scans (**d**). Donor IDs: 205 (**a**,** b**), 207 (**c**, **d**), 185 (**e**, **f**).

**Figure 12.**
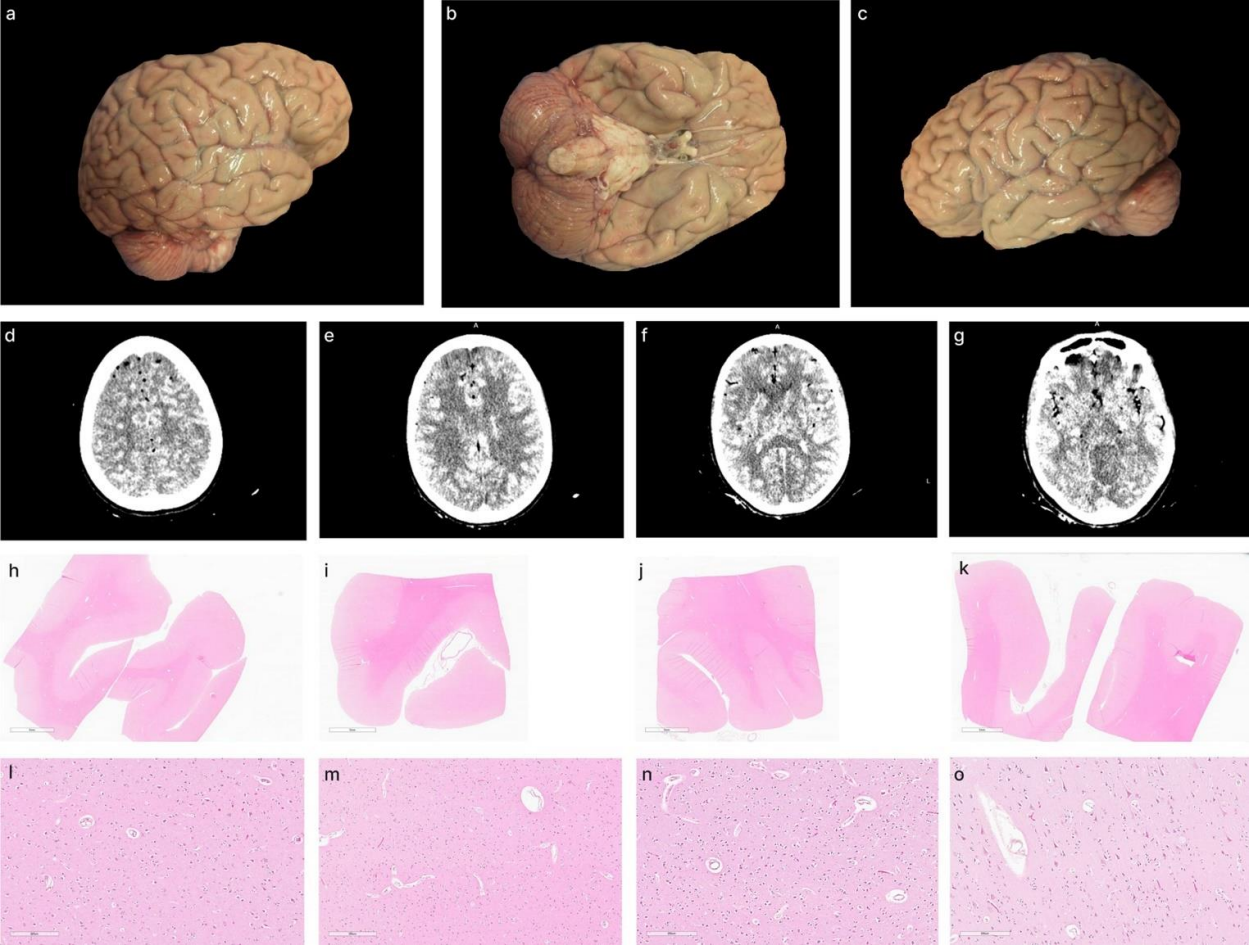
Representative gross examination, CT scan, and histology findings in a single brain. This brain was assessed a perfusion quality grade of 2 in all regions across all three modalities. Gross examination images: right lateral view (**a**), inferior view (**b**), left lateral view (**c**), and coronal CT images (**d–g**). H&E histology from the right frontal cortex (**h**, zoom in **l**), right temporal cortex (**i**, zoom in **m**), left temporal cortex (**j**, zoom in **n**), and left frontal cortex (**k**, zoom in **o**). Donor ID 107, PMI of 36.5 hours. Scale bars: 6 mm (**h**, **i**, **j**, **k**) and 200 μm (**l**,** m**,** n**,** o**).

Qualitatively, we found that in many cases, there was a spatial correlation between the perfusion profiles visualized via gross examination and those detected on CT scans (**Figure 11**). We also provide a representative example of perfusion correspondence across all three modalities: a brain from a 54-year-old donor with a PMI of 36.5 hours, which exhibited similar perfusion quality grades throughout the brain as measured by gross examination, CT imaging, and histology (**Figure 12**).

**Figure 13:**
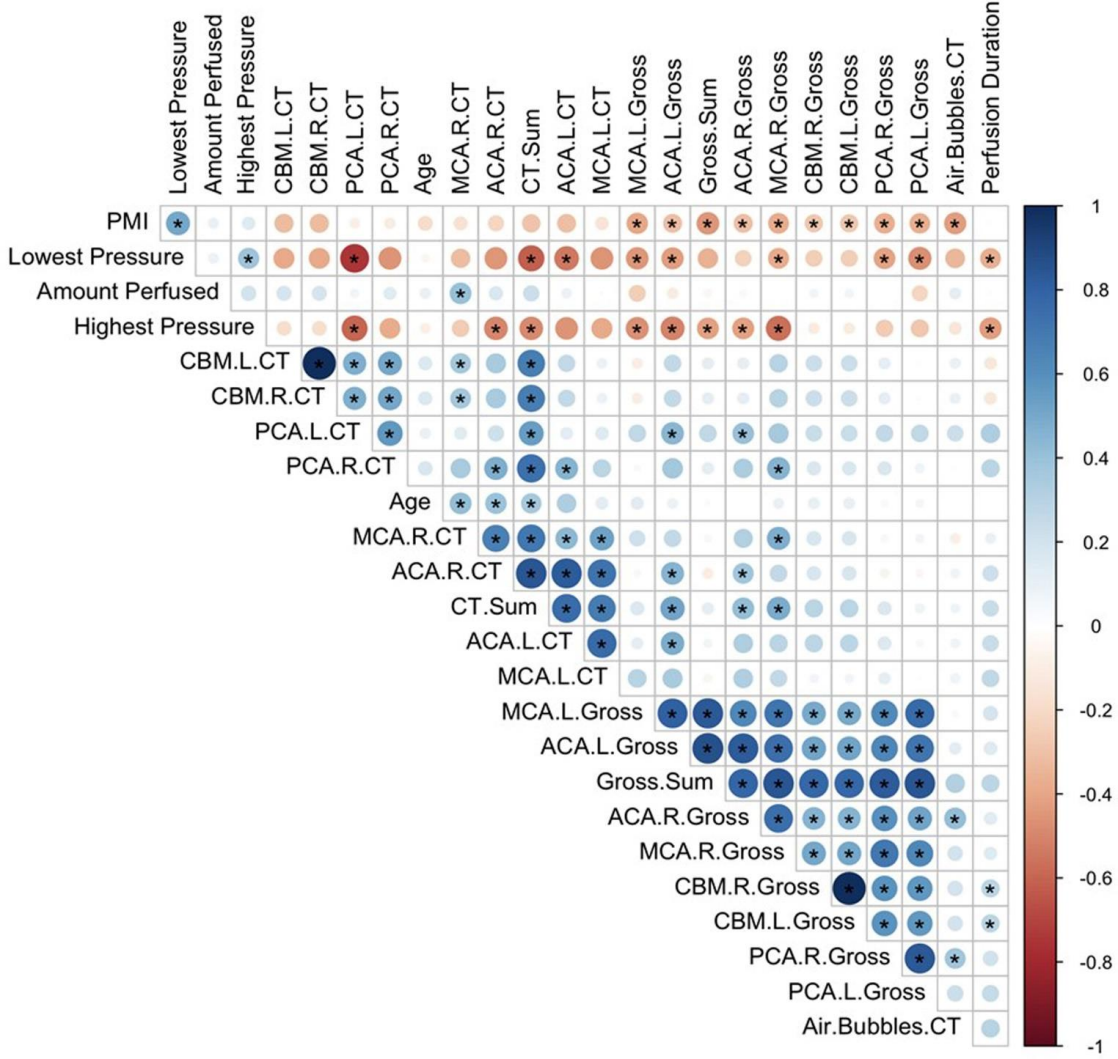
Correlation matrix of perfusion quality metrics across brain donors. Pearson correlation coefficients between perfusion quality assessments (gross examination and CT imaging), technical parameters (lowest and highest perfusion pressures recorded, total perfusate volume, and donor characteristics (age, postmortem interval (PMI)). Perfusion quality was assessed in multiple areas: the vascular territories supplied by the anterior cerebral artery (ACA), middle cerebral artery (MCA), and posterior cerebral artery (PCA), as well as the cerebellum (CBM), with left (L) and right (R) hemispheres evaluated separately. Sum scores correspond to the aggregate perfusion quality grades across all evaluated regions for both gross examination (Gross.Sum) and CT imaging (CT.Sum). Air.Bubbles.CT indicates the grade for the extent of air bubbles visible on post-perfusion CT scans. Circle size and color intensity indicate correlation strength, with blue representing positive correlations and red representing negative correlations. Asterisks (*) denote statistically significant correlations (p < 0.05, unadjusted).

To more comprehensively compare the perfusion quality scores across different methods, we next measured the correlations between quality scores and other relevant variables (**Figure 13**). We also created metrics of whole-brain perfusion quality for gross examination and CT scans by summing the grades across all assessed regions (insufficient cases with histology were available to perform similar quantitative correlations). We found no significant correlation between the sum of gross image grades and the sum of CT scan grades (correlation coefficient r = 0.12, p = 0.57, degree of freedom df = 23), which may be due in part to the limited number of samples available for comparison. However, the grades for gross examination and CT scores did have a significant positive correlation in several individual regions, such as the left ACA area (r = 0.48, p = 0.0096, df = 26), although notably these p-values were not adjusted for multiple comparisons. Regarding PMI, there was a significant negative correlation between the PMI and the sum of the gross examination grades across areas (r = –0.45, p = 0.0004, df = 57), while the correlation between the PMI and the sum of the CT scan grades across areas was negative without reaching statistical significance (r = –0.29, p = 0.11, df = 29).

Regarding other technical variables, several correlations stand out. First, we found that the PMI was significantly correlated with the lowest recorded perfusion pressure (r = 0.50, p = 0.002, df = 32). We usually initiated flow at a consistent, relatively low rate, and the lowest recorded perfusion pressure is generally the perfusion pressure at this initial flow rate. Therefore, this result likely indicates lower vascular resistance in brains with a shorter PMI, which may reflect less time for the accumulation of factors that impair perfusion. The lowest recorded perfusion pressure also had a significant negative correlation with the sum of CT perfusion grades (r = –0.61, p = 0.002, df = 32), indicating that, under the same procedural protocol, the eventual quality of perfusion may be predicted to some extent based on the initial pressure reading at the start of the procedure. Notably, the grade for the extent of air bubbles observed on CT images was also significantly negatively correlated with PMI (r = –0.43, p = 0.015, df = 30). A shorter PMI may be associated with more air bubbles because the same factors that prevent adequate perfusate flow may also the introduction of air bubbles into the brain.

### Comparison of syringe and pump-based perfusion

We compared two commonly used methods for driving perfusate flow: (a) a peristaltic pump and (b) manual syringe injection. A number of previous studies have performed postmortem perfusion via syringe injection in the isolated cephalon (Mignucci-Jiménez et al., 2024; Pérez-Cruz et al., 2024) or whole brain (Smirnov et al., 2023). The syringe method has some advantages, for example being less resource-intensive and allowing manual assessment of pressure based on resistance felt during injection. In contrast, the pump method provides more continuous flow and the possibility to use higher pressures. We used peristaltic pump-driven perfusion in 61 cases and manual syringe injection in 16 cases. We found no significant difference between the two methods in the sum of perfusion quality grades on the gross examination images (13.2 for pump and 13.3 for syringe, t-test p-value = 0.97) or CT images (12.7 for pump and 13.0 for syringe, t-test p-value = 0.84). Similarly, there was no significant difference in the extent of air bubbles on CT images (1.45 for pump and 1.50 for syringe, t-test, p-value = 0.92). Among cases using the cephalon isolation approach, there was also no significant difference in the proportion of cases with herniation (24 % or 12/50 cases for pump, 14.2 % or 2/14 cases for syringe, two proportions z-test p-value = 0.68). These data suggest that the simpler syringe-based method may be a useful option when laboratories do not have access to a pump, although further research is needed to corroborate this finding.

## Discussion

In this study, we assessed perfusion quality across a sample of donated brains that were perfusion-fixed prior to immersion fixation. Our results highlight significant variability in perfusion quality both within and across brain specimens. The assessment methods we employed – gross examination, CT imaging, and histology – were partially correlated, but not perfectly, supporting their use as complementary approaches to evaluate perfusion outcomes. Consistent with the existing literature, we also found that the perfusion quality as assessed by gross examination images decreases with longer PMIs. Naïvely, one might expect that fluid perfused into the arteries should follow a physiological path through the capillaries, with balanced hydrostatic and oncotic pressure gradients controlling limited exchange of fluid across vessel walls, before the majority effluxes through the IJVs. However, we found that the physiology of postmortem perfusion clearly differs from *in vivo* circulation, indicating the need to study it as a distinct phenomenon.

One of the main differences between postmortem mechanical perfusion and *in vivo* circulation is the regional heterogeneity of perfusion quality, which we observed across all perfused specimens. The underlying reasons are unclear and likely multifactorial. *In vivo*, active compensatory and autoregulatory mechanisms help to maintain uniform perfusion. In contrast, the postmortem brain lacks these homeostatic mechanisms, leading to heterogeneous perfusion impairment across the brain. One contributing factor may be the uneven accumulation of clots that obstruct fluid flow through different vascular territories, which could be self-reinforcing. Specifically, areas that receive more initial perfusate may become progressively cleared of intravascular debris, while vascular territories with no initial perfusion remain obstructed and continue to accumulate factors that impair flow over time.

Another aspect of postmortem perfusion is the risk of edema, which we also noticed in previous cases using *ex situ* perfusion fixation (McKenzie et al., 2022). To mitigate the risk of edema and associated herniation, we began adding mannitol to the perfusate, which was included in the fixative perfusate for the majority of our cases. This approach was based on evidence that the osmotic concentration of the buffer in fixation solutions primarily determines its effective osmotic pressure (Pallotto et al., 2015). Although some members of our team subjectively thought that mannitol might slightly mitigate the risk of edema, we did not have enough observations about brain volume changes or other parameters with and without mannitol to assess this quantitatively. Nevertheless, mannitol clearly did not completely prevent edema and herniation. One potential explanation is that mannitol is not a large enough molecule to increase effective osmotic concentration when the BBB is damaged. Previous data indicates that in regions where the BBB is severely damaged, mannitol can leak through tight junctions into the brain parenchyma rather than creating the intended osmotic gradient in the blood vessels, negating its beneficial effects (Videen et al., 2001; Liu et al., 2022).

Air bubbles are another important variable to consider in postmortem perfusion. In liver transplantation, air bubbles can be introduced during organ procurement, reducing the extent to which the organ is perfused, and can be mitigated by avoiding the exposure of arteries to air (Liu et al., 2010; Izamis et al., 2014). We previously demonstrated via neuroimaging that *ex situ* perfusion-fixation of the human brain can introduce air bubbles (McKenzie et al., 2022). One potential concern with the isolated cephalon approach is that it might introduce air bubbles that hinder perfusion (Bolliger et al., 2018). The dose of air bubbles is likely important, as very small amounts of air seem both difficult to avoid in mechanical perfusion and unlikely to cause significant problems. One study on the perfusion of the isolated cephalon does not describe air bubbles being introduced or causing problems following a 10-minute-long period before carotid artery cannulation and perfusion, despite extensive imaging (Vrselja et al., 2019). Numerous other studies on the isolated cephalon or brain have also demonstrated that the adequate perfusion of such specimens is in principle possible (Lee et al., 1968; Gilboe, 1982).

In this study, we found that air bubbles were frequently visible on CT scans following perfusion, to varying degrees, but that the burden of air bubbles was not correlated with perfusion quality. Some of the variability in air bubbles may be attributable to differences in the amount of residual blood in vessels following cephalon isolation, which could affect how easily air enters the brain. Procedural differences may also contribute, as we did not consistently implement measures to mitigate the introduction of air emboli. Such measures to could include inverting the cephalon, clamping or irrigation of vessels prior to their cannulation, and performing initial retrograde perfusion to displace air already trapped in the proximal arteries. One potential confound is that air bubbles could also be introduced after perfusion, for instance when cannulae are removed. In future research, this technical confound could be minimized by maintaining perfused fluid within the blood vessels using plastic clamps that do not interfere with CT imaging.

One potential avenue for future research is the use of a washout solution prior to fixative perfusion. Although we perfused a washout solution in our first two perfusion cases, in subsequent cases we immediately perfused fixative solution. Our findings suggest that a washout solution may not be necessary, as we were able to effectively remove blood from the vessels with fixative alone. The duration required for formaldehyde to act as a chemical fixative is much longer than the time during which the fluid physically pushes out intravascular material, suggesting that theoretical concerns about red blood cells or clots being fixed in place are unlikely to affect perfusion outcomes, consistent with previous literature (Pease, 1964). One potential advantage of a washout solution is that it could incorporate chemicals, such as detergents, specifically designed to break down intravascular material that impedes perfusion, thereby potentially improving the quality of subsequent fixative perfusion (Tobin, 1970; Bradbury and Hoshino, 1978; Grabherr et al., 2008; Frigon et al., 2024). However, if such chemicals are to be used in a brain banking context, it would be essential to test whether they alter cellular structure. For example, detergents can damage lipid membranes, making them a potential double-edged sword: while they may help perfusion quality by facilitating the removal of blood clots, they also could compromise the visualization of cellular structures.

We acknowledge that perfusion fixation will almost certainly remain a specialized technique, rather than a routine practice in most brain banks. Instead, immersion fixation is likely to remain the standard method, as it has several advantages, including lower cost, higher reproducibility, and the possibility to flash freeze the other hemisphere unfixed. Additionally, immersion fixation may provide adequate preservation quality on its own, depending on the research application (Garrood et al., 2025). However, in the right research context, perfusion fixation may prove particularly useful. For example, a brain mapping study that requires high-fidelity preservation of cellular architecture across regions, such as light-sheet-microscopy-based reconstruction of neural circuits, may benefit from an intact brain with more uniform fixation throughout. Perfusion may also be useful for applications other than accelerating the process of fixation, such as mapping the vasculature with angiography, brain clearing, or distributing cryoprotective agents (McKenzie et al., 2024). The benefits of perfusion are particularly likely to be realized if the right brain donors are chosen, such as those with low PMI and a minimal agonal state. Furthermore, if methods can be developed to improve the quality of postmortem brain perfusion, then the technique could become more reliable and therefore more useful in applications requiring uniform and reproducible perfusion across the brain.

There are several limitations to our study. First, our analysis was qualitative or semi-quantitative rather than fully quantitative, which limits our ability to draw precise statistical conclusions about the relationships between perfusion variables and outcomes. Future studies incorporating more quantitative image analysis, as well as expanding the sample size, would strengthen these findings. Second, our perfusion methods were refined iteratively over the course of the study, introducing variability that may have confounded some of our comparisons. While this iterative approach was necessary for the development and optimization of the protocol, it limits our ability to isolate the effects of specific procedural variables. Third, we did not grade the severity of the agonal state of the donors prior to their death, as we often lacked sufficient information to do so. This is a limitation because the agonal state and cause of death likely have a substantial impact on perfusion quality.

Finally, another limitation concerns the potential variability in the concentration of iohexol contrast agent across specimens. This variation may have affected the perceived intensity of regional perfusion in CT images, potentially confounding our assessment of perfusion quality. Although iohexol does not cross vessels with intact BBB in most circumstances, it can traverse a compromised BBB in the context of ischemia (Mariajoseph et al., 2024). However, the BBB clearly does not fully break down immediately after cardiac arrest (Du et al., 2022). Because postmortem BBB integrity is expected to deteriorate progressively with increasing PMI, the iohexol distribution likely represents a combination of contrast agent confined within blood vessels and contrast agent that has penetrated into the parenchyma through a compromised BBB. The rate and extent of this penetration likely varied among cases with different PMIs, adding another layer of complexity to the interpretation of contrast distribution. While this is not a major confound for our primary analysis of the presence versus absence of perfusion in different parts of the brain, it does limit our ability to make quantitative comparisons of perfusion intensity across brains.

## Conclusions

The quality of human brain specimens is crucial for their adequate study, which in turn is essential to better understand the mechanisms of neurobiological disorders and improve our treatments for them. Existing methods for tissue preparation are often inadequate, and mechanical perfusion is one proposed way to improve the quality (Beach et al., 198; McFadden et al., 2019). In this study, we investigated the quality of perfusion across the brain using a single perfusion-fixation protocol. We found that in some cases, especially those with relatively shorter PMIs, mechanical perfusion successfully reached at least a subset of blood vessels throughout the brain. However, in other cases, perfusion quality was relatively poor, with the perfusate failing to reach most regions of the brain. In a significant minority of cases, perfusion also caused brain edema, leading to herniation and requiring interruption of the perfusion to prevent further tissue damage. We also found that the PMI is correlated with our perfusion quality assessment methods, but far from perfectly so, suggesting that other factors related to the condition of the brain at the time of perfusion also play a major role in determining perfusion quality. Further optimization of perfusion protocols may facilitate more rapid and uniform fixation of the brain and also performing other types of tissue preparation in the postmortem brain. Additional research in this area may also clarify the mechanisms of perfusion impairment after ischemia and enable the testing of methods to mitigate such impairment in human brains.

## Author contributions

J.S.S., K.F., J.F.C., and A.T.M. conceptualized the study. E.T. and C.D.S. performed light microscopy experiments. M.G., A.K., and A.T.M. performed data analysis. A.T.M. wrote the initial draft of the manuscript. All authors reviewed the manuscript and approved the final manuscript.

## Data availability

Whole slide image data can be accessed in a public repository on Zenodo, available here: https://zenodo.org/communities/mechanicalperfusioninbrainbanking. Code and data used for data analysis are available at https://github.com/andymckenzie/Perfusion_quality.

## Conflict of interest statement

Macy Garrood, Alicia Keberle, Gabriel Taylor, Jordan Sparks, and Andrew McKenzie are employees of Sparks Brain Preservation, a non-profit brain preservation organization.
